# Postoperative testicular metastasis in early to mid-stage gastric adenocarcinoma: a case report and literature review

**DOI:** 10.3389/fonc.2025.1654010

**Published:** 2025-09-10

**Authors:** Yuanshun Huang, Mengshuang Xu, Yaoyu Zhang, Yuan Xue, Lijun Wang, He Huang, Wei Li, Shadan Li

**Affiliations:** ^1^ Department of Urology, The 945th Hospital of Joint Logistics Support Force of Chinese People’s Liberation Army, Ya’an, Sichuan, China; ^2^ Department of Gastroenterology and Respiratory Medicine, The 945th Hospital of Joint Logistics Support Force of Chinese People’s Liberation Army, Ya’an, Sichuan, China; ^3^ Department of Urology, The General Hospital of Western Theater Command, Chengdu, Sichuan, China; ^4^ Department of General Surgery, The 945th Hospital of Joint Logistics Support Force of Chinese People’s Liberation Army, Ya’an, Sichuan, China

**Keywords:** gastric adenocarcinoma, testis, epididymis, immunohistochemistry, metastasis, case report, AI radiotherapy, AI chemotherapy

## Abstract

This study reports a case of a 59-year-old male who primarily presented with a testicular mass accompanied by pain, ultimately diagnosed as testicular metastasis from gastric adenocarcinoma. Over three years earlier, the patient underwent radical gastrectomy for gastric adenocarcinoma, followed by eight cycles of S-1 and oxaliplatin chemotherapy. On February 10, 2025, the patient presented to our hospital with a history of a left testicular mass that had persisted for over a year. He experienced recurrent, palpable pain in the right scrotal and inguinal regions. Contrast-enhanced computed tomography (CT) revealed a cystic and solid mass in the left testis; it displayed heterogeneous density, with punctate and linear enhancement within the parenchyma on contrast scans, showing a “fast in, fast out” pattern, while no significant enhancement was observed within the cystic lesion. Consequently, a left radical orchiectomy was performed, and histopathology confirmed metastatic gastric adenocarcinoma. This case provides new data for the clinical recognition of rare metastatic patterns of gastric cancer, highlighting the necessity of considering and investigating its clinical features, potential pathogenesis, and effective strategies to further prevent metastasis.

## Introduction

Gastric cancer ranks as the third leading cause of cancer-related mortality worldwide and the fifth most common cancer, accounting for the fourth highest number of cancer deaths globally (1–3). The epidemiological distribution of gastric cancer shows significant regional variation. Overall, the incidence remains high in Asia, with China, South Korea, and Japan leading the world in both incidence and mortality rates. Notably, the male population in these countries is disproportionately affected, with specific data indicating an incidence rate of 43.9 per 100,000 in Chinese males, 39.7 per 100,000 in South Korean males, and 48.1 per 100,000 in Japanese males (based on every 100,000 male population) (2). Pathologically, more than 95% of gastric cancers are adenocarcinomas (4). In contrast, primary adenocarcinomas of the testis and epididymis are relatively rare, and metastasis from gastric adenocarcinoma to these sites is exceedingly uncommon. Unlike the more frequently observed ovarian metastasis of gastric cancer (known as Krukenberg tumor), metastasis to the male reproductive system is extremely rare, with only a few cases reported worldwide (5–8). Cases involving simultaneous metastasis to both the testis and epididymis are extremely rare. This case involves a patient with early to mid-stage gastric adenocarcinoma who developed metastases to the testis and epididymis more than three years after surgery. This article provides new case data for the clinical understanding of rare metastatic patterns of gastric cancer, whose clinical features and pathogenesis have important research value, and the case reveals that it is necessary to think about and explore its clinical features, potential pathogenesis, and how to further prevent metastasis with effective measures.

## Case presentation

A 59-year-old male patient presented to our facility on February 10, 2025, with a chief complaint of a left testicular mass that had persisted for over a year. The patient reported the incidental discovery of a pea-sized swelling in the left testicle approximately one year prior, which was intermittently accompanied by mild discomfort and pain. There was no associated skin erythema, ulceration, hematuria, or pyuria. The patient initially self-administered antibiotics (specific agents unspecified), which provided temporary symptom relief; however, the aforementioned symptoms recurred periodically. Over time, the mass exhibited progressive enlargement, now approximately the size of a quail egg, with a notable increase in pain severity and limited response to antibiotic therapy.

The patient’s medical history includes the excision of a left ankle joint cyst performed at an external facility over a decade ago (details unspecified) and a ligation procedure approximately 20 years prior (details unspecified). About three years ago, the patient presented to an external facility with complaints of upper abdominal pain and discomfort. Endoscopic examination of the stomach revealed a large ulcer at the corner of the stomach, with a biopsy indicating poorly differentiated adenocarcinoma (signet ring cell carcinoma), pending exclusion of other diagnoses. On December 14, 2021, the patient underwent a laparoscopic distal gastrectomy with gastric-jejunal anastomosis and laparoscopic adhesiolysis for intestinal adhesions under general anesthesia. Intraoperative findings included adhesions between the omentum and the upper abdomen, as well as to the liver. A 3 cm ulcerative carcinoma was identified at the gastric angular region, exhibiting full-thickness invasion. Enlarged lymph nodes were observed near the common hepatic artery, near the left gastric artery, near the splenic artery, and in the peripylorus and perihepatic duodenal ligament areas. Postoperative pathology (December 20, 2021) confirmed a moderately to poorly differentiated adenocarcinoma of the distal stomach, infiltrating the entire gastric wall. The tumor measured 2.5 cm by 2.5 cm, with evidence of neural invasion within the lesion; no vascular invasion was noted. Immunohistochemistry results showed tumor cells positive for PIS2, MSH2, MSH6, MLH1, CKp; occasional positivity for CDX-2 and Villin; CK20 negative; CK7 positive; Ki-67 proliferation index approximately 65%; HER2 negative; SALL4 negative; hepatocyte marker negative; CEA positive. No tumor infiltration was observed at the resection margins. Lymph nodes within the gastric perivisceral fat: 2 out of 8 nodes exhibited metastatic involvement. Other lymph nodes (Group 3: 1 node; 12V group: 2 nodes) showed no evidence of metastasis.

### Final diagnosis

Moderately differentiated gastric adenocarcinoma (pT3N1M0, stage IIB), mismatch repair proficient (pMMR), HER2 negative, consistent with early to intermediate-stage gastric carcinoma. The genomic profiling indicates a potentially high sensitivity to platinum-based chemotherapeutics, with a comparatively reduced risk of adverse effects from 5-FU/capecitabine, irinotecan, and taxanes. Detected targeted therapy-associated genetic alterations include TP53 p.R248W and MET amplification, while variants of uncertain clinical significance comprise CDK4 p.R181Q and JAK2 p.L697H.

The patient underwent adjuvant chemotherapy with the SOX regimen during cycles 1 through 8 postoperatively: oxaliplatin 150 mg intravenously on day 1 combined with tegafur/gimeracil/oteracil (Tegio capsule) 50 mg orally twice daily from days 1 to 14, administered every three weeks. During the final chemotherapy cycle, due to chemotherapy-induced myelosuppression and moderate anemia, granulocyte-colony stimulating factor (G-CSF) was given to promote leukocyte recovery. Given the severity of anemia and the patient’s inability to tolerate combined modality therapy, monotherapy with the Tegio capsule 50 mg orally from days 1 to 14 every three weeks was continued, resulting in an uneventful chemotherapy course. From February 21 to March 25, 2022, the patient received definitive radiotherapy targeting the gastric carcinoma region: CTV 4500 cGy in 25 fractions at 180 cGy per fraction. The radiotherapy was well tolerated. During close follow-up over the next three years, no evidence of tumor recurrence has been observed.

Physical examination shows normal external genitalia development, with evenly distributed pubic hair and intact skin surface free of ulcers, scars, or fistulas. The urethral meatus is non-erythematous and free of discharge. Palpation of the left scrotum reveals an enlarged testis measuring approximately 3×4 cm, characterized by hardness, limited mobility, and adhesion between the testis, epididymis, and spermatic cord, with indistinct boundaries and tenderness upon pressure. An elongated, protruding mass is noticeable along the spermatic cord in the left inguinal region, without significant overlying skin erythema or ulceration; the spermatic cord appears markedly thickened, extending proximally toward the internal inguinal ring. The mass size remains unchanged when in the supine position. The right testis, epididymis, and spermatic cord are unremarkable on palpation.

Laboratory test results showed normal serum β-human chorionic gonadotropin (β-HCG) levels (less than 2.0 IU/L; reference values are 0 to 5.0 IU/L). Alpha-fetoprotein (AFP) is measured at 1.58 IU/mL (reference values are 0 to 6.13 IU/mL). Lactate dehydrogenase (LDH) activity is 195 U/L (reference values are 120 to 250 U/L). Carbohydrate antigen 19-9 (CA 19-9) is elevated at 163.56 U/mL (reference values are 0 to 27.5 U/mL). Carcinoembryonic antigen (CEA) is 9.41 ng/mL (reference values are 0 to 4.1 ng/mL).

### Ancillary examination

**Figure 1:**
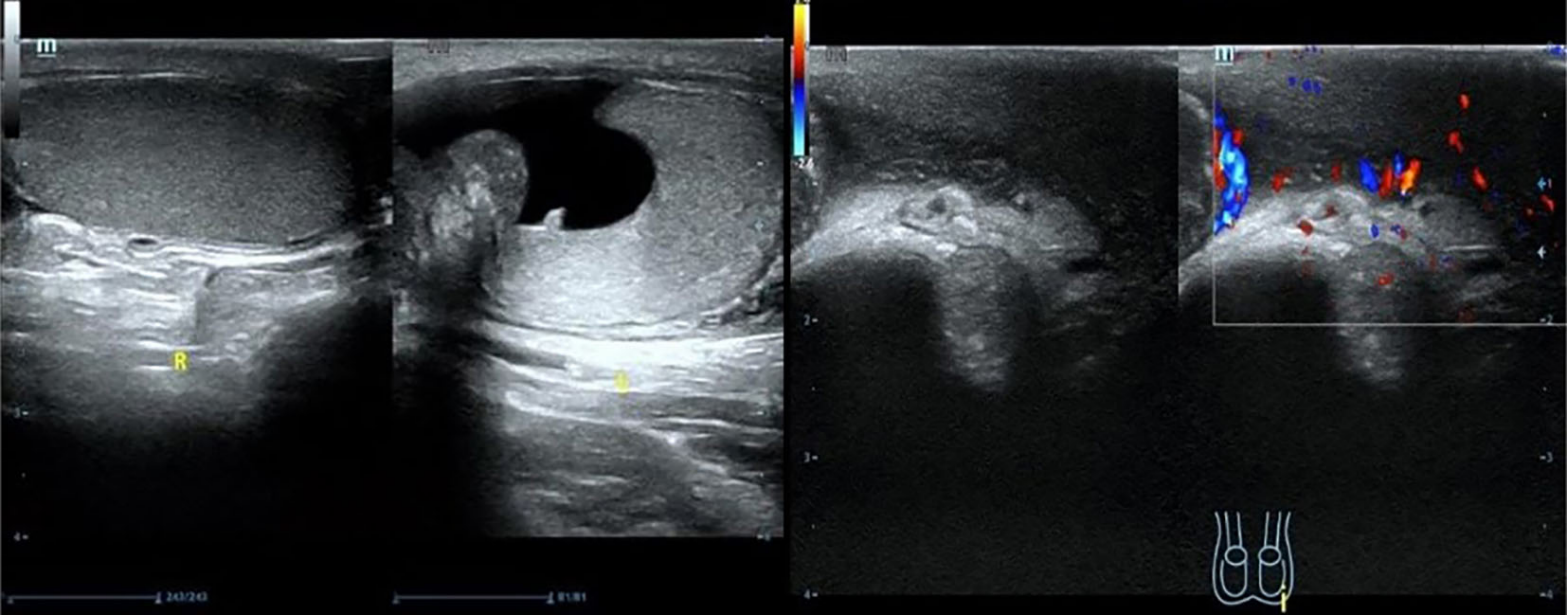
Scrotal ultrasound.

**Figure 2:**
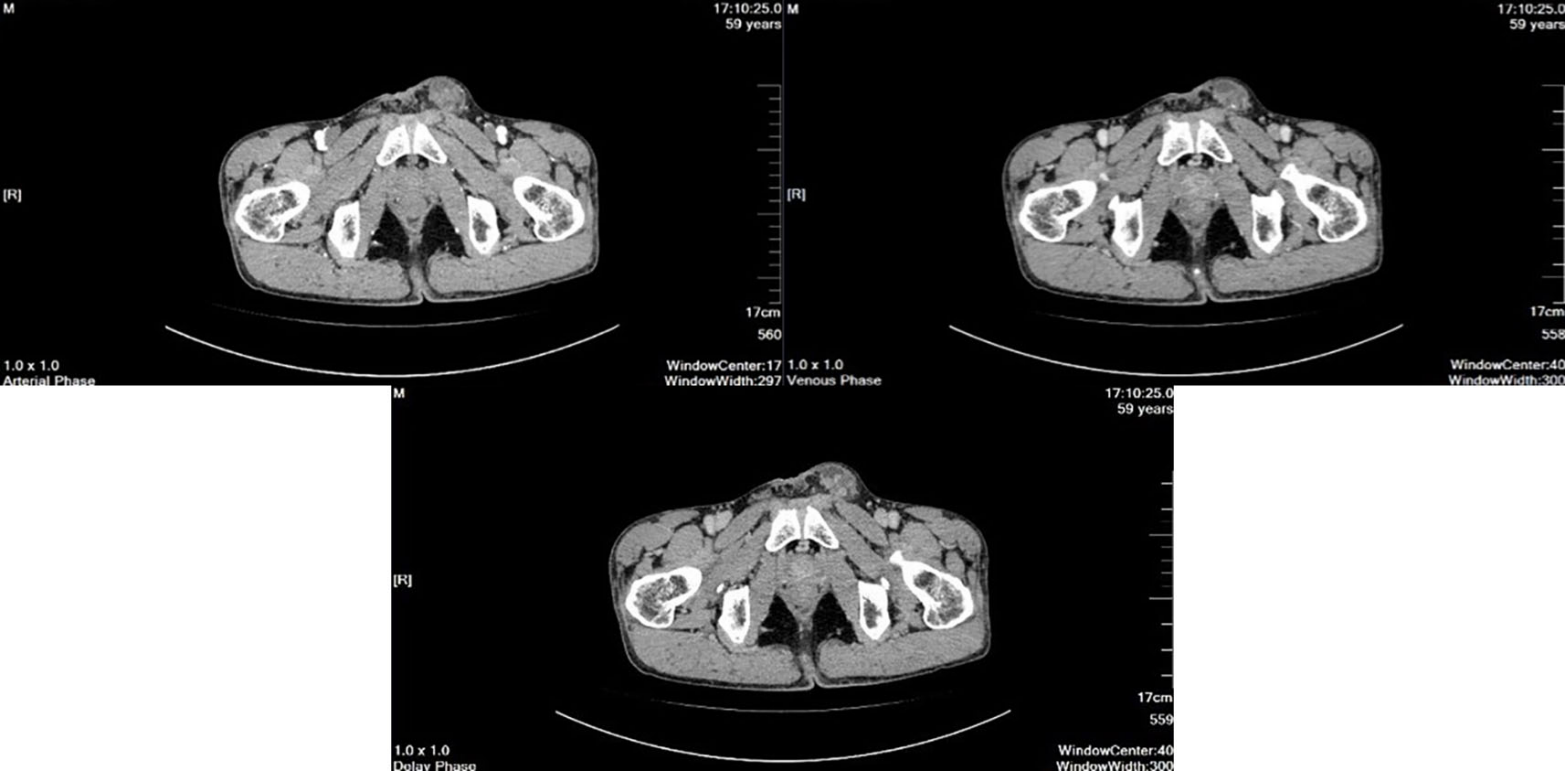
The enhanced computed tomography (CT).

Scrotal ultrasound revealed that “the left testis was in the shape of ‘reverse C’, with abnormal morphology, the left epididymis had uneven echogenicity and rich blood supply, the left testis had an anterior liquid dark area, and the left spermatic cord was thickened, with uneven echogenicity and a slightly rich blood supply(**Figure 1**)”. The enhanced computed tomography (CT) scan revealed “cystic solid occupancy of the left testis: uneven density of the left testis, point-stripe enhancement in the parenchyma, showing a ‘fast-in-fast-out’ pattern, and no obvious enhancement in the cystic foci” (**Figure 2**).

As a malignant tumor was suspected in the preoperative auxiliary examination, the patient underwent a left radical orchiectomy via the inguinal route under lumbar-rigid anesthesia.

**Figure 3:**
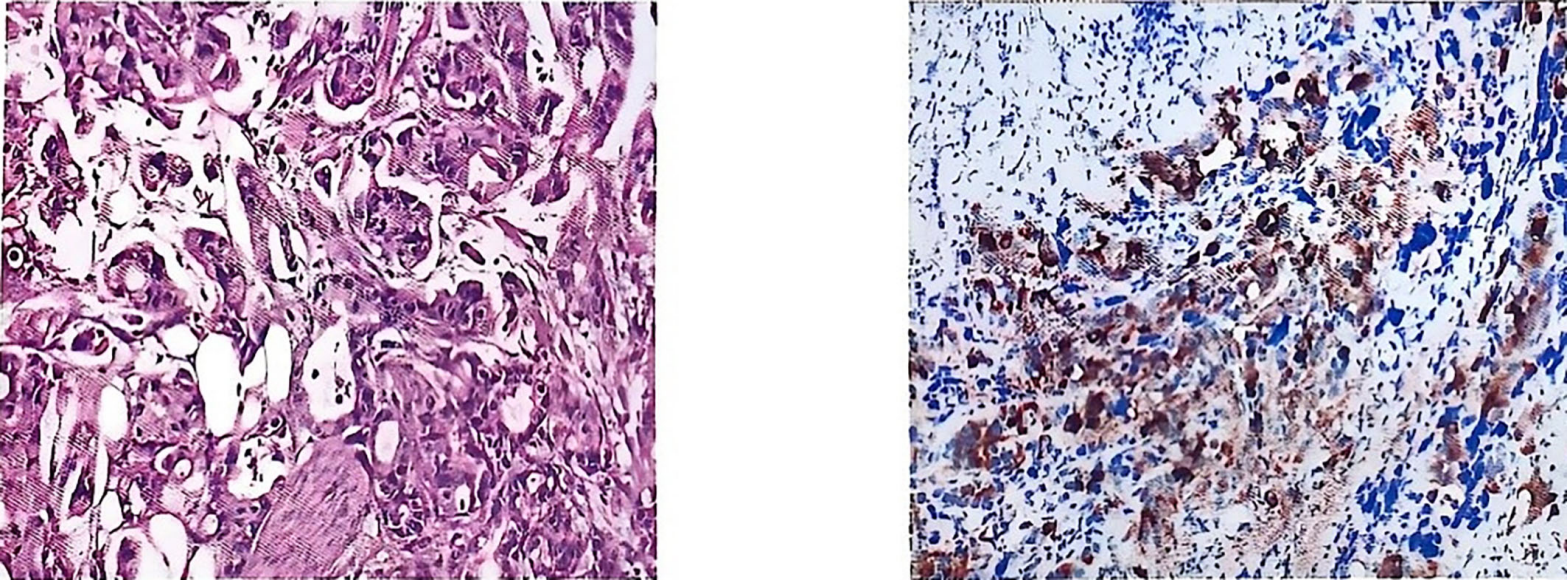
Postoperative pathology.

Postoperative pathology was consistent with metastasis of gastric adenocarcinoma. Pathological diagnosis: (left side) infiltration of poorly differentiated tubular adenocarcinoma in the testis and epididymis, with the maximum diameter of the tumor being approximately 4.0 cm; nerve bundle invasion (+), vascular invasion (+); no cancerous cells were observed in the margins of the surgical incision; the combination of the immunophenotyping examination results suggested the possibility of upper gastrointestinal tract origin, so please correlate with the clinical history. Immunomarkers: MUC5AC (+), CK7 (+), CK20 (+), CDX-2 (foci +), CEA (+), HER2 (-), TTF-1 (-), Ki-67 (+, about 50%) (**Figure 3**).

No cancer cell infiltration was found at the edge of the surgical site. Due to severe adverse effects of chemotherapy and radiotherapy (e.g., hematotoxicity and gastrointestinal reactions) after the last surgery, the patient was physically unable to endure the discomfort again and therefore only agreed to symptomatic treatment and close follow-up. The patient was followed up after six months with progression-free survival.

## Discussion

Gastric cancer generally has a poor clinical prognosis, which is mainly attributed to the fact that it often progresses to an advanced stage by the time of diagnosis, while early diagnosis can significantly enhance therapeutic outcomes (4). The most common metastatic target organs for this disease are the liver, peritoneum, lungs, and bone marrow. Metastases to the liver, lungs, bones, peritoneum, and regional lymph nodes are frequently observed in advanced cases, and these metastatic signs often indicate a poor prognosis. This emphasizes the importance of early diagnosis and intervention (2). The testes, epididymis, and spermatic cord are uncommon sites of metastasis and are typically observed in advanced stages (9).

Patients with gastric cancer in China typically exhibit the characteristics known as “three highs and one short”: high incidence, low early detection rate, high mortality rate, and short survival time. There is a significant disparity in the postoperative recurrence rate by stage: approximately 14% for early gastric cancer, and up to 60% for advanced gastric cancer. Notably, around 70% of patients with progressive gastric cancer experience recurrence or metastasis within two years after radical surgery, with abdominal implantation metastasis (34.9%) being the most common. Studies have confirmed that advanced gastric carcinoma-peritoneal metastasis (AGC-PM) is the leading cause of death in patients with advanced gastric cancer (10). Awareness, screening, healthcare, and healthy lifestyles for gastric cancer are crucial to diminishing its global impact (2).

According to existing studies, the mechanism of gastric cancer metastasis to the testis and epididymis has not been clarified; early literature considered the hematogenous route of metastasis (11–13). However, studies conducted after 2008 have generally questioned the clarity of their transfer pathways (9, 14–17). At currently, the following hypotheses exist regarding the metastasis of malignant tumors to the testis (14): (1) Lymphatic metastasis (9, 14): It includes conventional lymphatic metastasis and retrograde lymphatic diffusion, and there is bidirectional metastasis (stomach-testis mutual metastasis). (2) Anatomical channel diffusion hypothesis: body cavity implantation and metastasis (similar to the Krukenberg tumor model) extending directly through the blood vessel wall or unclosed sheath process (18). (3)Hypothesis of hematogenous diffusion (19): Multipathway spread can include direct diffusion and retrograde venous diffusion or embolization, as seen with renal and adrenal tumors that spread along the internal spermatic vein. (4) Direct infiltration (18): Such as a malignant tumor of the epididymis and spermatic cord (5). Tumor dormancy activation hypothesis (16, 20): The late recurrence of the primary tumor after treatment may be related to the reactivation of long-term dormant tumor cells in distant organs. The incidence of testicular metastasis is less than 2.5% of testicular malignant tumors, and its rarity is attributed to multiple anatomical barriers (white membrane wrapping, low temperature environment, blood-testis barrier, and hanging position) (14, 17). Prostate cancer (32%) and lung cancer (19%) were the primary focuses. Notably, distinguishing between primary and secondary testicular tumors can be challenging (13). Metastasis of malignant tumors to the epididymis is rare, and concurrent metastasis to both the testis and epididymis is even rarer than a primary tumor (12, 19, 21).

This patient resembles previously reported cases (14). Considering our patient’s previous vasectomy, the palpable, painful mass associated with syringomyelia and possible occlusion of the bilateral inguinal ducts may have hindered the spread of tumor cells through the vaginal eminence. The pathology revealed invasion of the testicular sheath wall layer and the chorda tympani, and the risk of metastasis by implantation was considered high. However, lymphatic spread could not be ruled out due to the presence of syringomyelia, suggesting that lymphatic drainage might be impaired, which could lead to retrograde lymphatic spread.

It is important to note that primary testicular cancer is most common in men aged 15 to 35 years, whereas metastatic testicular cancer is more prevalent in men aged 50 to 60 years and older. Additionally, testicular masses occurring in this age group are more likely to be metastatic (22). As described in this case and in the literature, the clinical manifestations and tumor markers often lack specificity. The primary imaging feature is a predominantly cystic-solid mass in the testis and adjacent tissues. Distinguishing between metastatic and primary testicular tumors on imaging can be challenging; thus, it is necessary to combine imaging results with abdominal/pelvic CT and gastrointestinal scintigraphy to exclude metastatic foci. Ultimately, the final diagnosis relies on histopathology (23).

In this case, the patient exhibits initial symptoms of a testicular mass and inguinal pain, which aligns with the report (19). The most common signs of male genital metastasis include a mass in the scrotum or discomfort in the groin area, sometimes accompanied by syringomyelia and scrotal swelling. Because of their unusual symptoms, these conditions are often misdiagnosed as primary tumors of the male genital organs, hernias, epididymitis, or orchitis. Thus, it is necessary to consider the patient’s medical history comprehensively. For patients with a history of gastrointestinal malignant tumors, rare metastatic tumors of the reproductive system should not be overlooked (24). Although tumor markers and imaging methods such as ultrasound, CT scans, MRI, and PET-CT can provide valuable information for diagnosis, the exact differential diagnosis of tumors relies on biopsy procedures and histopathological analysis. While the diagnosis takes distant metastases into account, the fact that the condition is advanced does not imply that necessary surgical treatment should be forsaken (24). In cases where metastases are limited to the reproductive organs and are considered resectable, complete surgical resection combined with subsequent radiotherapy and chemotherapy can significantly improve survival outcomes.

Through in-depth analysis, the disease progression in this case led us to reflect on the effectiveness of existing diagnostic and therapeutic strategies for preventing rare metastases from primary tumors. Reviewing the diagnostic and treatment processes of this case, we found that although conventional radiotherapy is widely used, it has recognized limitations in treatment precision, toxicity management, and individualized optimization, which may potentially undermine its effectiveness in preventing metastasis. Notably, current medical technology is undergoing significant changes, and the integration of artificial intelligence (AI) in radiation oncology is advancing rapidly, showing transformative potential. Studies have confirmed that multifunctional AI models demonstrate good task execution and significant generalization capabilities in multicenter cohort data (25, 26). Specifically, the application of AI in radiation therapy (AI radiotherapy) is promising. For example, AI-automated contouring is expected to replace manual methods, saving significant costs and time, and allowing professionals to focus more on patient care (27). Incorporating AI in radiologic imaging and treatment planning enhances the accuracy of anatomical diagnostics and the effectiveness of various treatment plans, highlighting the transformative power of artificial intelligence in medical imaging (25). Meanwhile, AI-assisted chemotherapy regimen design (AI chemotherapy) has demonstrated potential for clinical application. Preliminary studies support the feasibility of using AI to optimize chemotherapy drug selection, calculate dosages, determine timing, and predict efficacy and toxicity. The main advantage lies in its ability to integrate multi-dimensional data, resulting in more accurate and personalized treatment decisions (28). Therefore, we hypothesized that if such AI chemotherapy strategies could be prospectively integrated and implemented in the postoperative adjuvant treatment phase, they might provide a new way to reduce the risk of rare postoperative metastases, including the type presented in this case, by enhancing the precision and efficacy of treatment. Of course, more prospective clinical studies are urgently needed to validate this speculation (28).

The intensive application of AI/ML (machine learning) technologies in radiation oncology and chemotherapy optimization is driving a transformation in treatment by enhancing accuracy, efficiency, and personalization. It plays a crucial role in patient selection, treatment planning, dosimetry, response assessment, and even drug design and tumor classification (29, 30). AI is emerging as a transformative tool capable of analyzing factors such as cancer type, stage, and patient characteristics to support personalized treatment strategies, including antibody selection, radioconjugate options, and dosage adjustments. This development opens up new possibilities for advancing precision cancer care (30).

In this study, we report a case of postoperative testicular epididymal metastasis from gastric cancer in a patient whose genetic testing revealed gene variants of unknown clinical significance, along with those related to targeted drug administration. Three years after the radical resection of the *in situ* tumor, this patient developed rare testicular epididymal metastases, which led us to propose the scientific hypothesis that these genetic variants of unknown significance may be potentially associated with the unusual metastatic properties of the tumor. A search in the GeneCards database found that the mutation site of CDK4p.R181Q was associated with gastric adenocarcinoma (the correlation score was low), but no related records of JAK2p.L697H were retrieved. Additionally, neither the CDK4p.R181Q nor the JAK2p.L697H mutation sites were identified in the core genome database related to urinary system tumors. Existing evidence indicates that the research data regarding this mutation site in specific tumor types (such as urinary system tumors) may not be fully represented, or that further investigation is needed. This study aims to serve as a reference for establishing future research pathways by systematically reporting this case: (1) Accumulating pathological data on similar rare metastasis cases; (2) Enhancing the long-term follow-up database of genetic test results; and (3) Exploring the correlation between genetic variant profiles and metastatic patterns. This research idea can provide important insights for subsequent clinical practice and scientific research: by systematically collecting and analyzing the genetic features of rare metastatic cases, it is expected to reveal the molecular mechanisms underlying the unique metastatic behaviors of tumors, ultimately leading to precise predictions and interventions for individualized treatment. It is worth mentioning that, because this case is only a case report, there is a lack of follow-up research to verify the correctness of the scientific hypothesis.

## Data Availability

The original contributions presented in the study are included in the article/supplementary material. Further inquiries can be directed to the corresponding authors.
